# Zika Virus Pathogenicity Versus Transmissibility. Comment on Roozitalab et al. Distinct Virologic Properties of African and Epidemic Zika Virus Strains: The Role of the Envelope Protein in Viral Entry, Immune Activation, and Neuropathogenesis. *Pathogens* 2025, *14*, 716

**DOI:** 10.3390/pathogens15010067

**Published:** 2026-01-08

**Authors:** Manfred Weidmann, Oumar Faye, Martin Faye

**Affiliations:** 1 Institute of Animal Hygiene and Veterinary Public Health, Leipzig University, 04109 Leipzig, Germany; 2 Institute for Microbiology and Virology, Brandenburg Medical School Fontane, 01968 Senftenberg, Germany; 3 Institut Pasteur de Dakar, Dakar 10000, Senegal

## Zika Virus Pathogenicity Versus Transmissibility

Interest in Zika virus (ZIKV, *Orthoflavivirus zikaense*) evolution and pathogenicity has attracted the attention of a wider circle of the research community ever since ZIKV emerged on the South American continent in 2015, after more or less island hopping across the Pacific Ocean from Southeast Asia on its way from Africa where it was first described in Uganda in 1947 [1]. The codon adaptation index of African ZIKV genomes to the human genomes of monkeys and humans is higher than in Asian ZIKV strains. The African genotype ZIKV strains therefore potentially efficiently replicate in humans, which may explain spillover of the African genotype ZIKV to humans in sylvatic transmission cycles but possibly also asymptomatic transmission in a peri-urban cycle. ZIKV strain MR766 actually shows comparably poor growth in vertebrate cells (Vero) and in mosquito cells in comparison to other African ZIKV isolates, which could be correlated with the presence of N-glycosylation motif NDI at Asparagine 154 of the E protein, which is deleted in strain MR766. It is therefore not a typical African strain as all others studied replicate much better and produce more infectious viral particles in mosquito cells than in vertebrate cells. This may be linked to the reduced pathogenicity in human cells than that induced by epidemic Asian genotype ZIKV, which shows long-term persistence, more consistent with clinical manifestations [2,3].

Recently, Roozitalab et al. [4] studied ZIKV E protein binding efficiencies of African ZIKV strain (MR766) and an epidemic Brazilian ZIKV strain (BR15). In comparison to strain BR15, strain MR766 exhibited enhanced viral binding to and destruction of neuronal SH-SY5Y cells and produced larger plaques and 1-log higher titers in Vero67 cells but induced a lower pro-inflammatory response in SH-SY5Y cells. Additional C-prM chimaera experiments confirmed the role of the E protein in neurosphere disruption, through strong viral attachment and replication. The C-prM region was found to influence infection efficiency and permissiveness, with both structural components contributing to the overall observed phenotype especially in the African C-prM-containing chimera. Since the least efficient replicating African strain ZIKV strain MR766 already yields remarkable SH-SY5Y infection results, the findings may actually be significant. We would like to point out that the efficient binding of the E protein of ZIKV strain MR766 to the neuronal SH-SY5Y cell receptors may reflect the higher codon adaptation to the human genome and thus co-evolution of the African strains with vertebrate hosts including humans. Codon adaptation as an indicator of replicative efficiency may have provided the space for E adaptation to the receptor.

Recent work has also provided evidence that the greater transmissibility of African lineage ZIKV strains compared to their Asian counterparts has a polygenic nature and may have prevented Asian lineage strains from achieving the same transmission efficiency as African lineage strains [5]. That African strain MR766 and epidemic strain BR15 are mechanistically different in terms of infection and permissibility to neuronal cells may indicate potential pathogenicity of African strains for humans. Currently, they have only been shown to be virulent in mice and have not been involved in large outbreaks.

Altogether, however, the adapted transmissibility of the Asian epidemic strains might be more important for the emergence of ZIKV in human health than the general pathogenicity they manage to induce. This evolutionary view, however, should not disregard the incidence and impact of the more severe congenital ZIKV syndrome and Guillain–Barré syndrome observed in ZIKV epidemics.
